# Anomalous transverse resistance in 122-type iron-based superconductors

**DOI:** 10.1038/s41598-018-37152-y

**Published:** 2019-01-24

**Authors:** Yangyang Lv, Yu Dong, Dachuan Lu, Wanghao Tian, Zuyu Xu, Wei Chen, Xianjing Zhou, Jie Yuan, Kui Jin, Song Bao, Shichao Li, Jinsheng Wen, Liviu F. Chibotaru, Tobias Schwarz, Reinhold Kleiner, Dieter Koelle, Jun Li, Huabing Wang, Peiheng Wu

**Affiliations:** 1 Research Institute of Superconductor Electronics, Nanjing University, Nanjing, 210023 China; 2 Beijing National Laboratory for Condensed Matter Physics and Institute of Physics, Chinese Academy of Sciences, Beijing, 100190 China; 3 Key Laboratory for Vacuum Physics, University of Chinese Academy of Sciences, Beijing, 100049 China; 4 University of Chinese Academy of Sciences, Beijing, 100049 China; 5 School of Physics, Nanjing University, Nanjing, 210023 China; 6 National Laboratory of Solid State Microstructures and Department of Physics, Nanjing University, Nanjing, 210093 China; 7 Theory of Nanomaterials Group, KU Leuven, Celestijnenlaan 200F, Leuven, B-3001 Belgium; 8 Physikalisches Institut-Experimentalphysik II and Center for Collective Quantum Phenomena in LISA, Universität Tübingen, Auf der Morgenstelle 14, Tübingen, D-72076 Germany; 9 Synergetic Innovation Center in Quantum Information and Quantum Physics, University of Science and Technology of China, Hefei, 230026 Anhui China

## Abstract

The study of transverse resistance of superconductors is essential to understand the transition to superconductivity. Here, we investigated the in-plane transverse resistance of Ba_0.5_K_0.5_Fe_2_As_2_ superconductors, based on ultra-thin micro-bridges fabricated from optimally doped single crystals. An anomalous transverse resistance was found at temperatures around the superconducting transition, although magnetic order or structure distortion are absent in the optimal doping case. With the substitution of magnetic and nonmagnetic impurities into the superconducting layer, the anomalous transverse resistance phenomenon is dramatically enhanced. We find that anisotropic scattering or the superconducting electronic nematic state related with the superconducting transition may contribute to this phenomenon.

## Introduction

For a low-dimensional superconductor, like an ultra-thin film, an anomalous transverse resistance (ATR) can often be observed as the temperature is lowered towards *T*_c_, thus the investigation of ATR will provide insight into the dynamics of the condensation of Cooper pairs. The origin of ATR for conventional superconductors was attributed to various effects like geometric asymmetry^1^, vortex motion^2,3^, or even an inhomogeneous distribution of superconductivity^4–7^. However, the ATR observed in the high-*T*_c_ copper oxides superconductor seems to have a more complex origin^8^. First, since antiferromagnetic order occurs in the under-doped state of these materials^9,10^, an anomalous Hall effect may contribute to the ATR in the absence of external magnetic fields. The anomalous Hall effect is generally due to the spontaneous magnetization in the ferromagnetic system^11,12^, spin-fluctuation^13^, side hops or skew scattering from magnetic impurities^14^, or even from topological effects (Berry curvatures)^15^. However, the anomalous Hall effect vanishes in a paramagnetic conductor, failing to explain the ATR in the optimally or over-doped cases of cuprates in the absence of magnetic order. Quite recently, an anomalous transverse voltage is reported on the under-doped La_2-*x*_Sr_*x*_CuO_4_ single-crystalline thin films^8^, and particularly, the in-plane angular-dependent ATR exhibits a sin(2*φ*) oscillation breaking the four-fold rotational symmetry of the lattice. The origin of this two-fold ATR was attributed to the anisotropic electronic state, namely, the electronic nematicity, which provides a promising path to understand the ATR in this case.

As another high-*T*_c_ family, the iron-based superconductors have a similar phase-diagram of superconductivity and magnetic phases as that of cuprates, thus the corresponding superconducting mechanism for both families should share some common behavior^16^. However, the Fe-based superconductors have five 3*d* bands which contribute to the Fermi surface, resulting in a rather complicated multiband structure. In the under-doped case, most of the crystals demonstrate an antiferromagnetic order at temperatures below the critical point of Curie temperature *T*_s_, and almost simultaneously, a symmetry breaking from C_4_ to C_2_ happens within the electronic structure^17–20^. The electronic nematic state is generally considered to be related to the structure, although the relation between the electronic ordering and structure transition is still a “Chicken and Egg Problem”^17,18^. Up to now, abundant researches have been carried out on the magnetic order and electronic nematic states in the iron-based superconductors, while the corresponding studies on the ATR still lack. Therefore, it is greatly important to explore the dynamics of electron pairing and transport, which yields to the origin of magnetic order and electronic nematicity.

**Figure 1:**
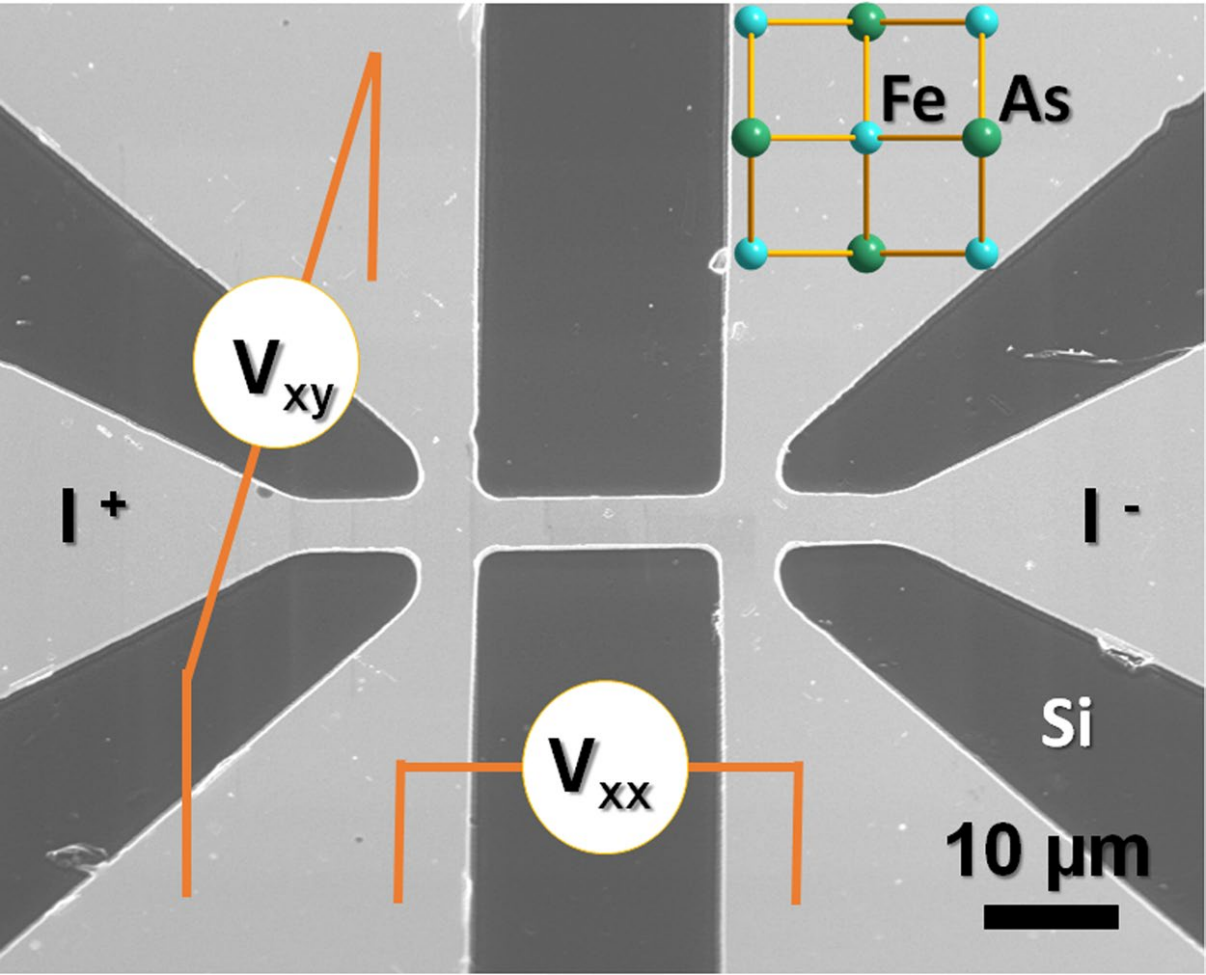
SEM image of a typical micro-bridge and electrodes for longitudinal resistance (*R*_xx_) and transverse resistance (*R*_xy_) measurements. The micro-bridge has a width of 5 *μ*m, length of 20 *μ*m, and thickness of 120 nm. The currents are applied along the Fe-As chains.

In this work, we investigated the in-plane transverse resistance of Ba_0.5_K_0.5_Fe_2_As_2_. This hole-doped 122-type compound was selected because high-quality single crystals are available. The transport measurements were performed on ultrathin single-crystalline micro-bridges, which are shown in Fig. 1. A pronounced ATR was found at temperatures around the superconducting transition, although the magnetic order is absent in the case of optimal doping. With the substitution of magnetic or nonmagnetic impurities into the superconducting layer, the ATR is significantly enhanced. The anomalous Hall effect, vortex motion, or electronic nematic state can hardly be regarded as the origin of the observed ATR, and a possible origin will be discussed.

## Results

### Anomalous Transverse Resistance

**Figure 2:**
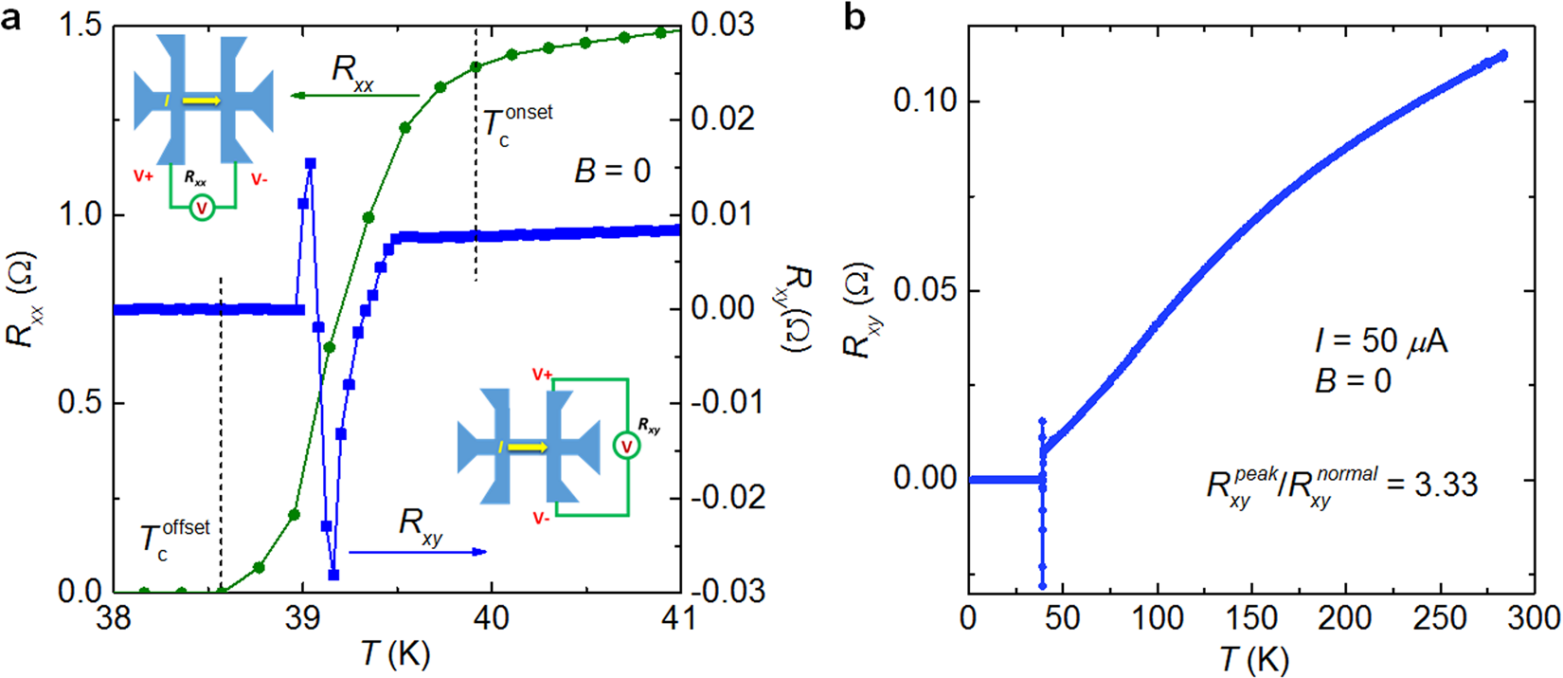
(**a**) Temperature dependence of *R*_xx_ and *R*_xy_ for BK micro-bridges. Inset schematic images indicate electrodes used for *R*_xx_ and *R*_xy_ measurements. (**b**) Temperature dependence of *R*_xy_ in the region from room to low temperatures. The applied current was 50 *μ*A for both measurements. No magnetic field was applied.

Figure 2 shows the temperature dependence of *R*_xx_ and *R*_xy_ for the BK micro-bridge. The midpoint of the resistive transition, as determined from *R*_xx_ is at 39.1 K, and the transition width is about 1.4 K. The values of *ρ*_xx_ (~12 *µ*Ω cm at 40 K) are about one order of magnitude smaller than bulk crystals^21,22^, indicating the high quality of the crystal and the improved measurement setup as described in previous work^23–25^. We emphasize that in the traditional four-probe measurement technique, a high current bias is often necessary to enhance the measurement signal, due to the extremely low resistivity and the large size of cross-section area. Consequently, heating effects and measurement errors may obscure the intrinsic transport properties of the crystals. For the micro-bridges used in this work, the resistance can be up to tens of ohm, being considerably higher than the interfacial contact resistance between the sample and the thin film gold electrodes (~0.1 Ω at room temperature). We note that the interfacial contact resistance has been well improved because of the *in-situ* fabrication and the annealing processes as introduced in a recent work^26^, by which the Schottky contact from the interface can be well eliminated.

*R*_xy_ vs. *T* exhibits a very different profile from *R*_xx_. In principle, *R*_xy_ should be completely zero, once the electrodes for the voltage measurements are perfectly symmetric. For the micro-device, however, an asymmetric structure often happens, resulting in a finite resistance *R*_xy_. For the present case, a weak resistance of about 0.008 Ω is observed at 40 K, which is two orders of magnitude less than *R*_xx_ (~1.39 Ω at 40 K). ATR peaks occur at temperatures below the *T*_c_-onset, which is the central finding of this work. The negative peak of *R*_xy_ at 39.1 K is about 3.33 times the value of the normal state resistance at 40 K (see Fig. 2(a)). Overall, *R*_xy_ vs. *T* exhibits two sign reversals. At the same time, for the temperature region up to room temperature the *R*_xy_ vs. *T* curve shows a similar profile as *R*_xx_ vs. *T* (see Fig. 2(b)), indicating that in the normal state *R*_xy_ is due to the asymmetric structure of the voltage electrodes. However, for the temperatures below the *T*_c_-onset, the dramatically different profile of the *R*_xy_ vs. *T* curve from those of *R*_xx_ vs. *T* suggests that the anomalous peaks should be related to the superconducting transition. We have measured more than ten samples, all of them demonstrate anomalous peak in the *R*_xy_(*T*) curves, while the value of the peaks is considerably different.

### Effect of Applied Current

**Figure 3:**
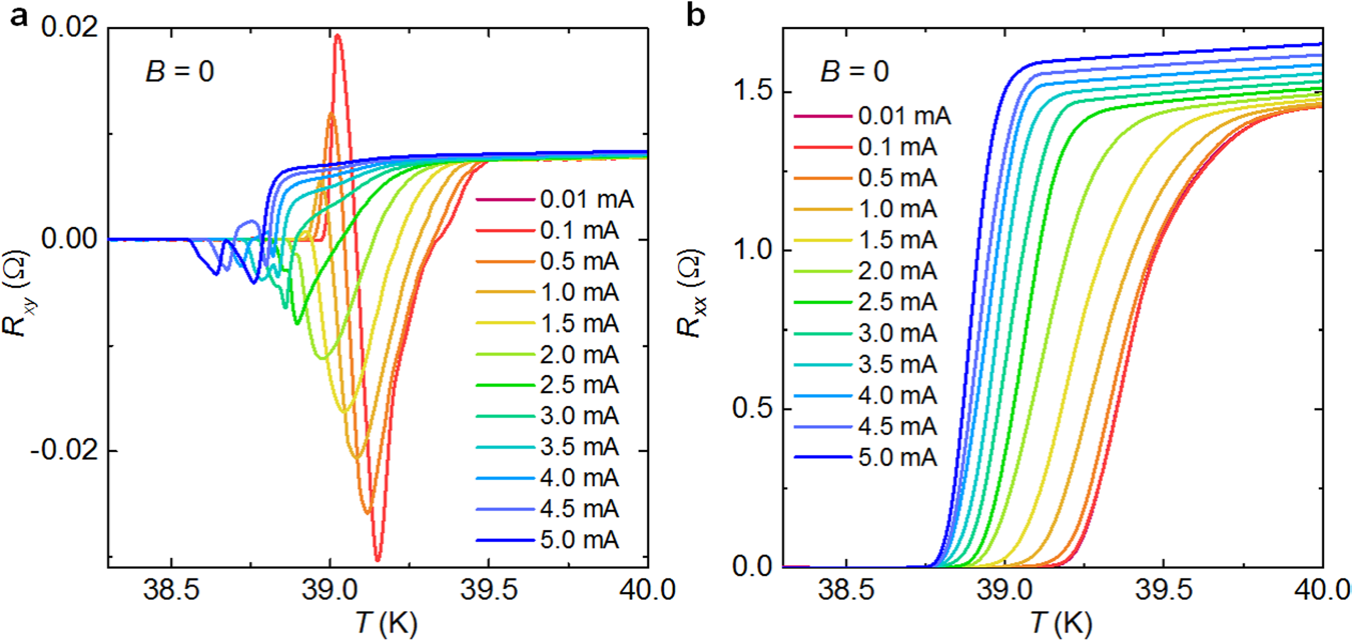
Temperature dependence of (**a**) *R*_xy_ and (**b**) *R*_xx_ for BK micro-bridges under different bias currents ranging from 0.01 mA to 5.0 mA.

To understand the link between the anomalous peaks and the superconducting transition, we applied various currents to suppress the superconductivity. Figure 3(a) demonstrates the temperature dependent *R*_xy_ under applied currents ranging from 0.01 to 5.0 mA. The anomalous peak is strong under weak current (0.01 mA) and is gradually suppressed by the applied current. Interestingly, the positive peak can be completely suppressed by a current of 2.5 mA, while the negative peak still exists for all currents. For comparison, the temperature dependence of *R*_xx_ under applied currents ranging from 0.01 to 5.0 mA is also given in Fig. 3(b). The current gradually suppresses the superconductivity as those of *R*_xx_ –*T* curves, while the anomalous peak is absent. It is worth noting that the normal resistance of *R*_xy_ is enhanced by the increasing current density, and such phenomenon was also found on the Ba(Fe_1-*x*_Co_*x*_)_2_As_2_ bulk crystals once the applied currents are up to the critical points^27,28^. Here, the current density is considerably high as about 1 MA/cm^2^ for *I* = 5 mA. The resistance enhancement is not due to the heating effect, because the *R*_xy_ –*T* curves can be completely repeated by increasing or decreasing the temperatures. Instead, a spatial variation of superconductivity may induce such effect^27,28^.

### Effect of Magnetic Fields

**Figure 4:**
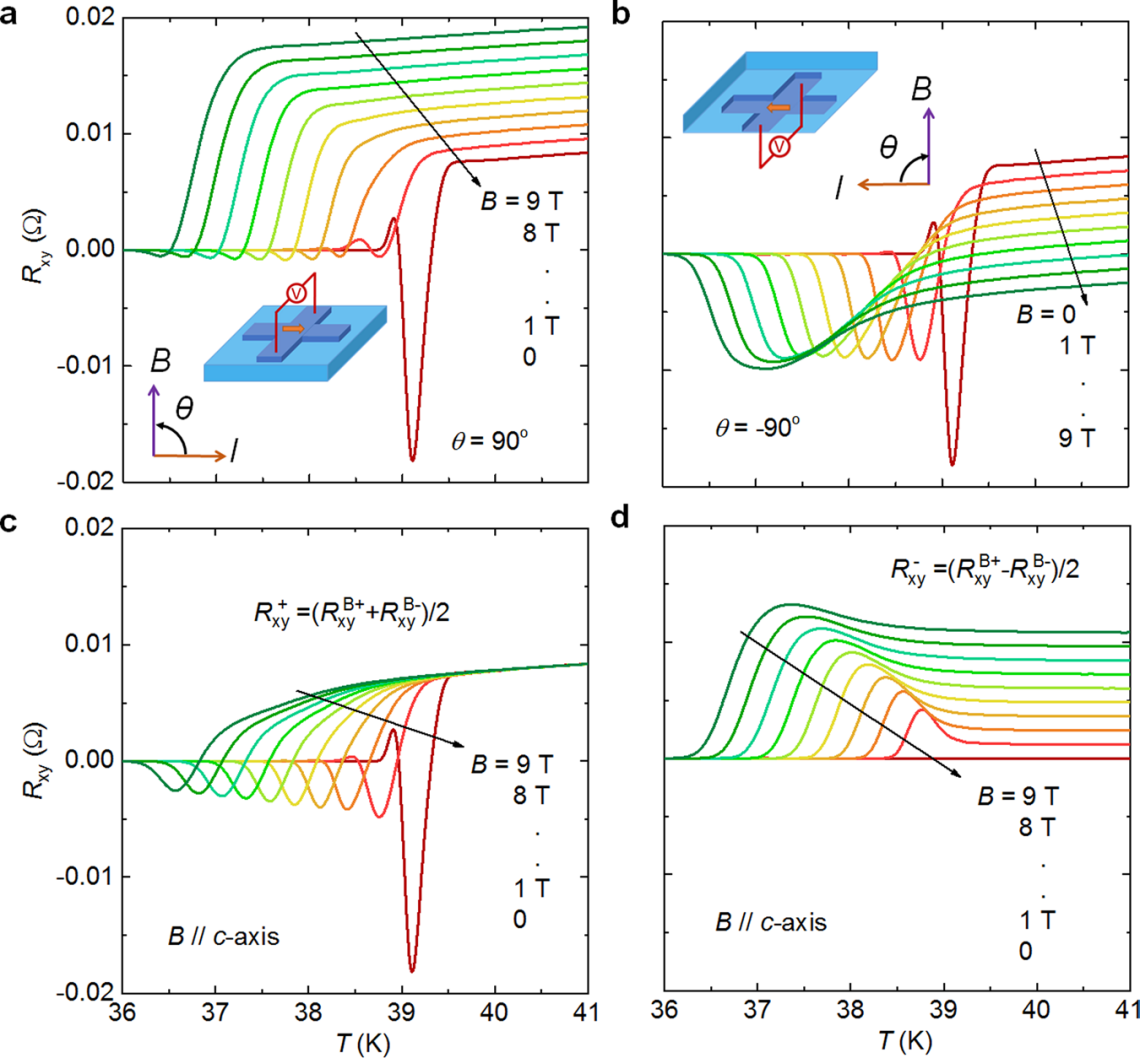
Temperature dependence of *R*_xy_ for BK micro-bridges for different out-of-plane magnetic fields between 0 and 9 T. In (**a**) the field was applied at an angle *θ* = 90° relative to the current direction. In (**b**) *θ* = −90°. (**c**) Magneto-resistance versus temperature, *R*_xy_ is calculated as ${R}_{{\rm{xy}}}^{+}=({R}_{{\rm{xy}}}^{{\rm{B}}+}+{R}_{{\rm{xy}}}^{{\rm{B}}-})/2$ to avoid the contribution of Hall resistance (Superscripts B+ and B− refer to the magnetic fields applied at *θ* = 90° and *θ* = −90°). (**d**) Hall resistance after eliminating the magneto-resistance: ${R}_{{\rm{xy}}}^{-}=({R}_{{\rm{xy}}}^{{\rm{B}}+}-{R}_{{\rm{xy}}}^{{\rm{B}}-})/2$.

Figure 4(a,b) give the temperature dependence of *R*_xy_ under out-of-plane magnetic fields between 0 and 9 T, applied at different angles *θ* relative to the direction of bias current. The negative peak in *R*_xy_ is suppressed dramatically, but this suppressing effect works weakly on the positive peaks. Since in magnetic fields the Hall effect will contribute to *R*_xy_, the magneto-resistance under the field along the *c*-axis (*θ* = ± 90°) is calculated as ${R}_{{\rm{xy}}}^{+}=({R}_{{\rm{xy}}}^{{\rm{B}}+}+{R}_{{\rm{xy}}}^{{\rm{B}}-})/2$ to eliminate the Hall contribution. Here, ${R}_{{\rm{xy}}}^{{\rm{B}}+}$ and ${R}_{{\rm{xy}}}^{{\rm{B}}-}$ are the magneto-resistance under positive (*θ* = 90°) and negative (*θ* = −90°) magnetic fields, respectively. In fact, ${R}_{{\rm{xy}}}^{+}$ has two contributions, which are the normal magneto-resistance Δ*R*(*B*) due to Lorentz force and the change in the transverse resistance ${\rm{\Delta }}{R}_{{\rm{T}}}(B)$, but it is clear to see that Δ*R*_T_(*B*) dominates. Figure 4(c) shows ${R}_{{\rm{xy}}}^{+}$ vs. *T* curves calculated from Fig. 4(a,b). Here, the positive peak is sensitive to the magnetic fields along the *c*-axis, while the negative peak is more resistive. For the ${R}_{{\rm{xy}}}^{-}=({R}_{{\rm{xy}}}^{{\rm{B}}+}-{R}_{{\rm{xy}}}^{{\rm{B}}-})/2$, there is no contribution from magneto-resistance or transverse resistance terms, thus basically the ${R}_{{\rm{xy}}}^{-}$ can be considered as Hall resistance. The ${R}_{{\rm{xy}}}^{-}$ vs. *T* curves demonstrate no anomalous peaks as shown in Fig. 4(d), suggesting that the anomalous peak is independent of Hall effect.

### Sample Geometry

**Figure 5:**
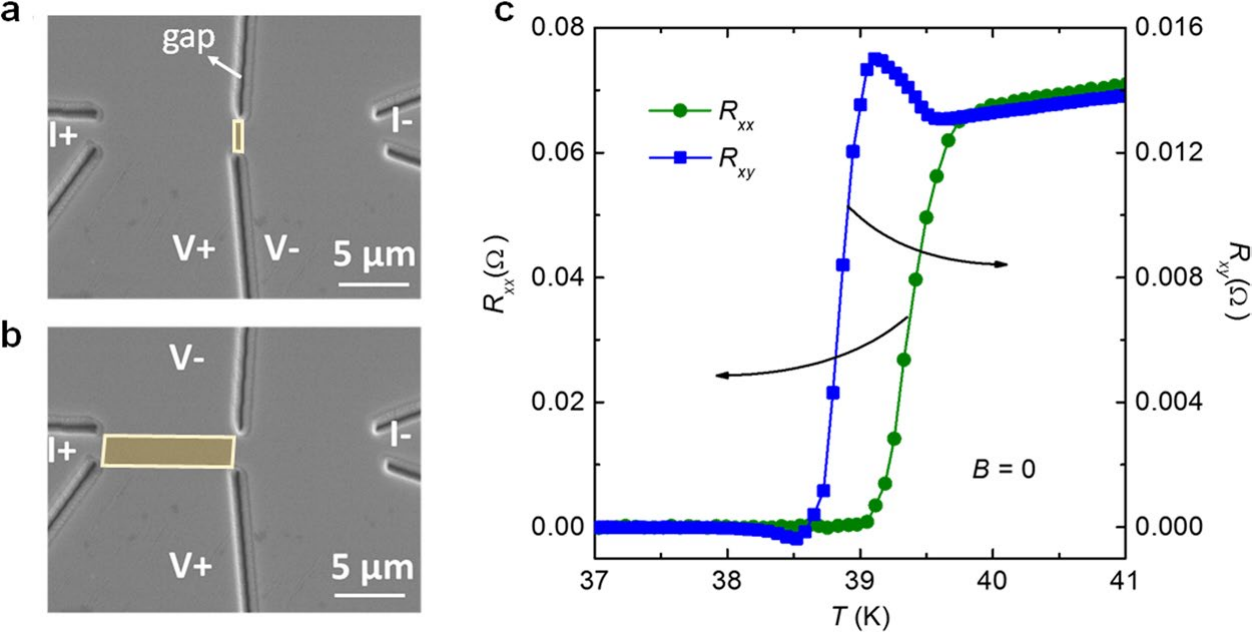
SEM images of the BK nano-bridges cut by focused ion beam milling. (**a**) The nano-bridge region for *R*_xx_ measurements, where the yellow rectangle is marked as the measurement region. (**b**) The measurement geometry for *R*_xy_ measurements marked in yellow rectangle as well. (**c**) Temperature dependence of *R*_xy_ and *R*_xx_.

To further investigate the effect of asymmetry on the voltage leads *V*+ and *V*−, we also fabricated a nano-scaled bridge by focused ion beam (FIB) milling technique as shown in Fig. 5(a,b). The length of the bridge is about 700 nm. Figure 5(c) gives the corresponding temperature dependent *R*_xx_ and *R*_xy_. Note that the anomalous peak for the *R*_xy_ vs. *T* curve also appears just below the *T*_c_-onset, while no such anomalous peak is visible in the *R*_xx_ vs. *T* curve, which is consistent with the micro-bridge samples as Fig. 2. Therefore, we can conclude that the anomalous peak is unlikely to arise from the geometry of samples. Some intrinsic effects related to the superconducting transition seem to be responsible for the anomalies.

### Impurity Doping Effects

**Figure 6:**
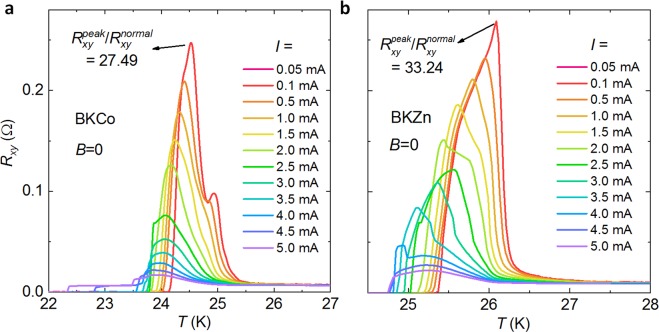
Temperature dependence of *R*_xy_ for the micro-bridges doped with (**a**) Co and (**b**) Zn impurities on the Fe-site of the superconducting layer Fe_2_As_2_.

Although the present Ba_0.5_K_0.5_(Fe,*M*)_2_As_2_ single crystals are of high quality in both crystalline structure and superconductivity^22^, the distribution of supercurrent can hardly be completely homogenous within the crystals, especially at the superconducting transition region. Thus, the effect of impurities or disorder should be seriously considered in the present micro- and even nano-scaled samples. To understand the impurity effects within the superconducting Fe_2_As_2_ layers, we substituted the Fe-sites by both magnetic Co and nonmagnetic Zn ions with a weak doping level of 2.5%, and measured the *R*_xy_ for the same micro-device geometry as the sample shown in Fig. 1. The temperature dependent *R*_xy_ for Ba_0.5_K_0.5_(Fe,*M*)_2_As_2_ doped with Co and Zn are shown in Fig. 6(a,b), respectively. The *R*_xy_ vs. *T* curves of both impurity-doped samples are quite similar: First, dramatically large anomalous peaks are observed under a low applied current of 0.01 mA, for which the peak value $({R}_{{\rm{xy}}}^{{\rm{peak}}}/{R}_{{\rm{xy}}}^{{\rm{normal}}})$ is about 33.24 for the Co-doped and 27.49 for the Zn-doped samples, being substantially larger than those of impurity-free samples (see Fig. 2). In fact, we have measured tens of samples, and the peak values for these samples vary from 10 to 100. Therefore, we can conclude that the substitution of impurity ions on the superconducting layer can enhance the anomalous transverse resistance.

**Figure 7:**
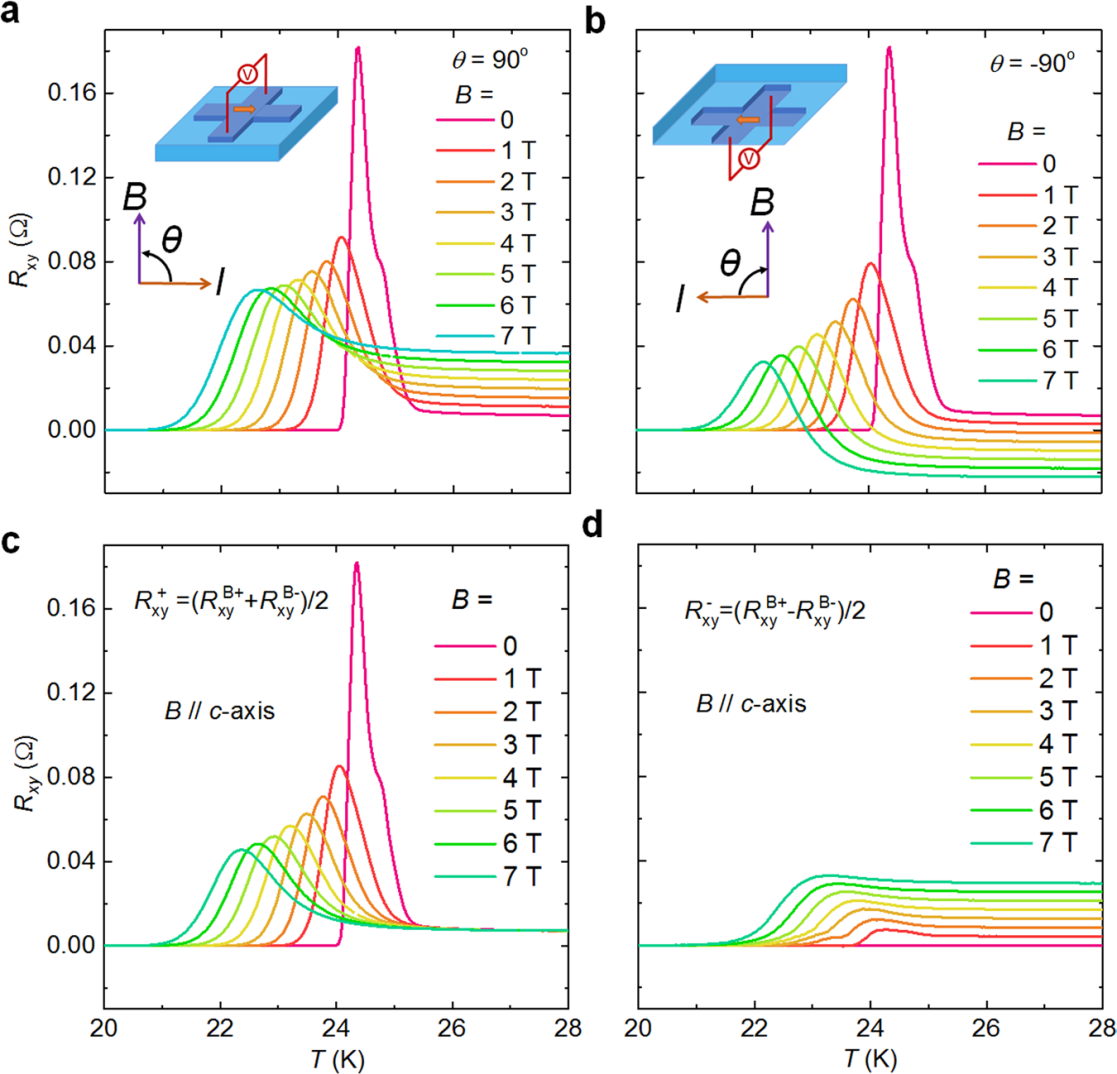
Temperature dependence of *R*_xy_ for Co-doped BK micro-bridges under magnetic fields for different angles with respect to current: (**a**) 90° and (**b**) −90°. (**c**) Temperature dependent magneto-resistance calculated from ${R}_{{\rm{xy}}}^{+}=({R}_{{\rm{xy}}}^{{\rm{B}}+}+{R}_{{\rm{xy}}}^{{\rm{B}}-})/2$ to avoid the contribution of Hall resistance. (**d**) Temperature dependent Hall resistance avoiding contributions from the magneto-resistance ${R}_{{\rm{xy}}}^{-}=({R}_{{\rm{xy}}}^{{\rm{B}}+}-{R}_{{\rm{xy}}}^{{\rm{B}}-})/2$.

The magnetic field-suppression effect on the anomalous peaks is in accordance with the impurity-free samples. Here, we take the Co-doped sample as an example. Figure 7(a,b) show the transverse resistance under magnetic fields along *θ* = 90° and −90°, respectively. The magneto-resistance ${R}_{{\rm{xy}}}^{+}=({R}_{{\rm{xy}}}^{{\rm{B}}+}+{R}_{{\rm{xy}}}^{{\rm{B}}-})/2$ is shown in Fig. 7(c). The Hall resistance has been fully eliminated, which can be confirmed by the magnetic field independent normal resistance, while the anomalous peaks are suppressed by the fields. However, for the Hall resistance ${R}_{{\rm{xy}}}^{-}=({R}_{{\rm{xy}}}^{{\rm{B}}+}-{R}_{{\rm{xy}}}^{{\rm{B}}-})/2$, the anomalous peak is absent (see Fig. 7(d)). Such profiles of the magnetic field-dependent magneto-resistance and Hall resistance are in accordance with the impurity-free samples, indicating the anomalous peak is a common property.

## Discussion

The anomalous peaks in the *R*_xy_ vs. *T* curves below the *T*_c_-onset may have various origins, which will be discussed in this session, including vortex motion, inhomogeneous distribution of the superconducting phase, and the intrinsic origins.

### Generally Possibilities

Since the anomalous peaks always occur within the superconducting transition region, vortex motion is one of the most likely possibilities for the origin, via the so-called vortex Hall effect. Hagen *et al*. found a sign reversal for the Hall voltage just below the superconducting transition of both high-*T*_c_ YBa_2_Cu_3_O_7_ and low-*T*_c_ Nb thin films^29^, which was attributed to vortex motion. However, the magnetic field is needed here to drive the vortex motion with a velocity *v*_L_. For the present experiments, the transverse voltage was observed under zero magnetic field, which requires a different model for explanation. Even for the Hall resistance as shown in Figs 4(d) and 7(d), the ATR is totally absent, being pronouncedly different from the vortex Hall effect.

In the case of zero field, the present transverse voltage may be due to the anomalous Hall effect (AHE)^30,31^. The magnetization of the sample itself can contribute to *R*_xy_ once the material transforms to the ferromagnetic state, resulting in AHE. Nevertheless, the present optimal-doped Ba_0.5_K_0.5_(Fe,*M*)_2_As_2_ is always in a paramagnetic state, which has been well confirmed by various measurements as neutron scattering^32^ and *μ*SR^33^. Therefore, one can hardly obtain an AHE effect in these samples.

On the other hand, the anomalous transverse voltage may be attributed to spin-orbit coupling, which can be of either an extrinsic origin due to disorder-related spin-dependent scattering of the charge carriers, or of an intrinsic origin due to a spin-dependent band structure of the conducting electrons^13^. The spin for AHE effects basically originates from electron-orbit coupling, which is a relativistic quantum mechanical effect^1,11^. However, the behavior of strongly current dependence and existence only in superconducting transition region can hardly be explained by the spin-orbit coupling model.

### Inhomogeneous Distribution

In the thin film systems, a non-uniform transport current can be considered as a possible explanation due to inhomogeneity of material or superconductivity^4–7^. Such ATR was widely found in the high-*T*_c_ and conventional superconductors, and the corresponding model have been proposed to explain at least part of the anomalous behavior. Since one can hardly avoid the problem of superconductivity spatial variation in thin films due to the island-like growth mechanism in most fabrication techniques, even for the molecular beam epitaxy method, it is rather challenge to grow a single-crystalline thin film with thickness up to few hundred nanometers. In the high quality Ba_0.5_K_0.5_Fe_2_As_2_ single crystals, although the external inhomogeneous can be considerably reduced and the superconducting Fe_2_As_2_ layer is a pure structure, the distribution of K ions in the Ba layer will induce inhomogeneous as well^34^. In a real compound, a finite number of micro-defects or impurities often exist in the lattice, which can be identified from the finite residual resistivity at 0 K (see more details in ref.^24^).

**Figure 8:**
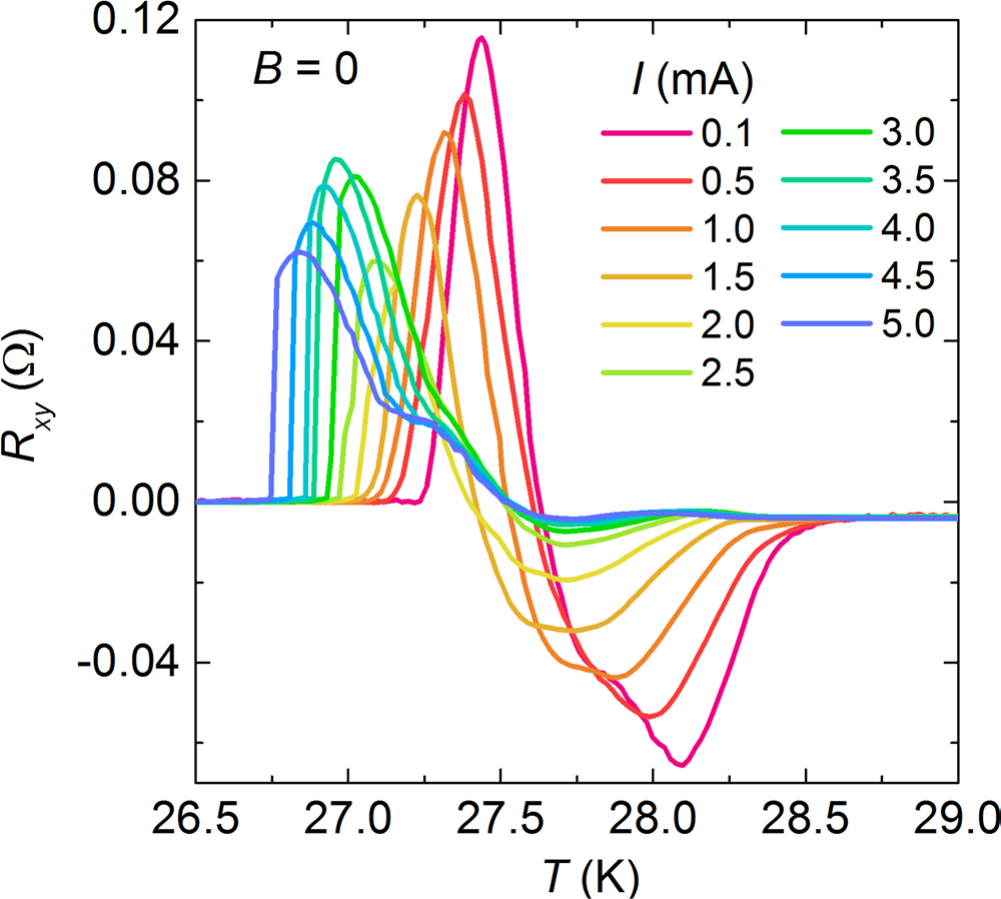
Temperature dependent *R*_xy_ for Co-doped BK micro-bridges under different currents ranging from 0.1 mA to 5.0 mA.

With substitution of atomic impurities into the superconducting Fe_2_As_2_ layers, the impurities are found to enhance the ATR phenomenon as introduced above. Substituting magnetic Co and nonmagnetic Zn onto the Fe-site in the Fe_2_As_2_ layers, the impurity ions can suppress the superconductivity within a region of a coherence length^22,24,25,35^, and then disorganize the superconducting distribution. Particularly, the impurity scattering centers are aligned with a certain direction, for instance the antiferromagnetic *a*-axis, resulting in highly anisotropic impurity states^36^. Such anisotropic scattering can induce the transport nematicity, i e. the ATR as shown in Fig. 6. Nevertheless, the impurity fails to explain the sign-reversal of the ATR as shown in Figs 2–5. Actually, the sign-reversal ATR happens in many samples. For instance, the Co-doped sample also demonstrates both positive and negative ATR below the *T*_c_-onset as shown in the *R*_xy_ vs. *T* curves in Fig. 8.

### Electronic Nematicity

A nematicity of the electronic state has been observed in both cuprates and iron-based superconductors^18–20,37–39^. Although the nematicity will shift in angle with the different doping levels, it breaks the *C*_4_ symmetry in all samples. Similar to the situation of the cuprate superconductors, the anomalous transverse voltage can be ascribed to this anisotropic electronic state^5^. The resistivity can be defined in *E* = *ρJ*, where *E* is the electric field, *J* is the applied current density, and the resistivity along the principal axes is $$\rho =(\begin{array}{cc}{\rho }_{{\rm{a}}} & 0\\ 0 & {\rho }_{{\rm{b}}}\end{array})$$ in matrix form. One can rotate the resistivity matrix as,

1 $${C}_{\varphi }\rho {C}_{\varphi }^{-1}=(\begin{array}{cc}{\rho }_{{\rm{a}}}{\cos }^{2}\varphi +{\rho }_{{\rm{b}}}{\sin }^{2}\varphi  & ({\rho }_{{\rm{a}}}-{\rho }_{{\rm{b}}})\cos \,\varphi \,\sin \,\varphi \\ ({\rho }_{{\rm{a}}}-{\rho }_{{\rm{b}}})\cos \,\varphi \,\sin \,\varphi  & {\rho }_{{\rm{b}}}{\cos }^{2}\varphi +{\rho }_{{\rm{a}}}{\sin }^{2}\varphi \end{array})$$

Due to the anisotropy of the electronic state, ${\rho }_{{\rm{a}}}\ne {\rho }_{{\rm{b}}}$, the off-diagonal term is non-zero. Therefore, as long as the applied current is not aligned with one of the principal axes, there will be a transverse voltage without magnetic field. For the present results, since the samples are in the optimally doped state, the structure distortion and the corresponding electronic nematic ordering along the *ρ*_a_ and *ρ*_b_ can be basically ignored. Thus, we can hardly apply the previous mechanism for cuprate superconductors onto the present results. Particularly, the present ATR phenomenon appears at temperatures just below the *T*_c_-onset, namely, with the occurrence of superconductivity. Therefore, another nematic ordering related to the superconducting transition should be taken into account for the ATR.

### Nematic Superconducting State

In our recent work, nematic ordering was found in the optimal-doped Ba_0.5_K_0.5_Fe_2_As_2_ superconductors at temperatures below the *T*_c_-onset, resulting in a superconductivity related nematicity^26^. Such nematicity demonstrates an electronic state ordering, and of course, will lead to anisotropic transverse resistance similar to the case of conventional nematic order in the normal state. However, the sample for the present measurements is rather small (few tens of micrometers), thereby, it is extremely hard to measure the angular-dependent ATR similar to the measurements on the (La,Sr)CuO_4_ thin films^40^. Despite the fact that serials of micro-bridges can be fabricated with different angles corresponding to current and lattice direction, it is almost impossible to obtain a systematic angular-dependent anomalous transverse resistance because one can hardly calibrate the longitudinal resistance contribution, which is due to asymmetries in the electrode configurations, as discussed in Section A. Anomalous Transverse Resistance in the Result part.

The support for such a scenario of anomalous transverse resistance is that it is manifested in the domain of nucleation of superconductivity, where the nematic superconducting state also appears. Moreover, as discussed in reference cited above, nematic superconductivity arises in sufficiently thin samples of Ba_0.5_K_0.5_(Fe,*M*)_2_As_2_ and is not expected to survive in bulk materials. This is fully in line with the phenomenology of the anomalous transverse resistance presented here, which gradually disappears with the increase of the thickness of the samples. What concerns the mechanism, we foresee two roots for the appearance of the anomalous transverse resistance in the nematic superconducting state, (i) via the anisotropic scattering from the nucleated superconducting domains in the normal/superconductor transition region and (ii) via the time-reversal symmetry breaking accompanying with the mixing of three components of the superconducting order parameter in the nematic superconducting state. To establish the relevant mechanism further experimental and theoretical studies will be needed.

## Methods

The synthesis method for Ba_0.5_K_0.5_Fe_2_As_2_ and Ba_0.5_K_0.5_(Fe,*M*)_2_As_2_ (*M* = Zn and Co) single crystals is described elsewhere^22^. Because of the high-pressure synthesis technique, the impurity ions (Zn or Co) could be homogenously distributed into the superconducting Fe_2_As_2_ layers to avoid disorders or other external defects. Here, the impurity-free optimally doped (Ba_0.5_K_0.5_Fe_2_As_2_) crystal was selected for which the antiferromagnetic order and the structure distortion are absent^26^. Ba_0.5_K_0.5_Fe_2_As_2_, nonmagnetic impurity Zn-doped (Ba_0.5_K_0.5_Fe_1.95_Zn_0.05_As_2_), and magnetic impurity Co-doped (Ba_0.5_K_0.5_Fe_1.95_Co_0.05_As_2_) superconductors are abbreviated as BK, BKZn and BKCo, respectively. The fabrication of micro-bridge is described in refs^23–25^. The samples were etched by ion beam milling for a few seconds to remove surface layers, and subsequently a gold film was deposited for electrodes via magnetron sputtering. Because fabrication steps were performed in different chambers, the samples were transported in a high-vacuum tube (<10^−10^ torr), resulting in a quasi *in-situ* fabrication process (AdNaNo-Tek Ltd.). The micro-bridges have a width (*W*) of 5 *μ*m, a length (*L*) of 20 *μ*m, and the thickness is confirmed from the longitudinal resistivity (*ρ*_xx_) at room temperature^26^. The *R*_xx_ and transverse resistance (*R*_xy_) were measured as a function of temperature in the Physical Properties Measurement System − 9 T, Quantum Design. Figure 1 shows a scanning electron microscope (SEM) image of a typical micro-bridge. Here the current was applied along the Fe-As bond direction.
